# Image derived input function applied in CBF Studies with [15O]water PET in an integrated MR-PET

**DOI:** 10.1186/2197-7364-1-S1-A30

**Published:** 2014-07-29

**Authors:** Jörg Mauler, Nuno André da Silva, Christian Filss, Heinz H Coenen, Hans Herzog, N Jon Shah

**Affiliations:** Institute of Neuroscience and Medicine, Forschungszentrum Jülich, Jülich, Germany; Department of Neurology, Faculty of Medicine, JARA, RWTH Aachen University, Aachen, Germany

To quantify CBF studies using [^15^O]water PET an input function must be provided, e.g. by means of arterial blood sampling. An image derived input function (IDIF) gained from the dynamic PET images may be a non-invasive alternative. The time-activity curve (TAC) of the blood can be obtained from a volume of interest (VOI) capturing the internal carotid artery (ICA).

Five healthy young volunteers were administered 555 MBq [^15^O]water four times each, by bolus intravenous injection. A 3 min PET listmode acquisition was started at the time of the injection (3TMR-BrainPET). Blood was automatically withdrawn from the radial artery to measure its TAC (TACblood, coincidence detector). A T1-weighted MPRAGE image (192x256x256 voxels, 1 mm^3^) was acquired. Applying corrections, the PET data were iteratively (OSEM3D) reconstructed (frame length 4s, 153x256x256 voxels, 1.25x1.25x1.25 mm^3^) and filtered (4mm 3D Gaussian). VOIs defining the ICAs in the MPRAGE image were transferred to the dynamic PET images to obtain an IDIF (TACcar). The dispersion and delay of TACblood were corrected (TACbloodcorr) using a one-compartment model and the whole-brain TAC. Cerebral blood flow (CBF) was quantified applying the autoradiographic ^15^O-water model using both TACbloodcorr and TACcar as input data.

Compared to TACbloodcorr, TACcar is affected by the partial volume effect (PVE) and the spillover (SP) from the temporal lobe. The PVE factor and the SP factor were 0.21±0.05 and 0.24±0.10, respectively. Global CBF differed by 8.4% ± 9.4%, when the TACcar after correction for PVE and SP was used as input data instead of TACbloodcorr.

The inherently coregistered, high-resolution 3TMR-BrainPET data are a suitable prerequisite for measuring the IDIF. After correction for PVE and SP, the IDIF may replace invasive measurement of ^15^O-activity.

